# CKAP2L Promotes Proliferation and Migration of Glioma Cells in a DIAPH3-Deficient Model

**DOI:** 10.3390/cells15211996

**Published:** 2026-10-31

**Authors:** Asma Mahdi, Arwa Aldaalis, Georges Chehade, Eva On-Chai Lau, Hend Salah Fayed, Ayman Al Haj Zen, Mohamed Aittaleb, Maisa Ismael Alkailani, Fadel Tissir

**Affiliations:** 1 College of Health and Life Sciences, Hamad Bin Khalifa University, Penrose House Building, Education City, Doha P.O. Box 34110, Qatar; asmamahdi.contact@gmail.com (A.M.); aaldaalis@hbku.edu.qa (A.A.); hend.fayed.91@gmail.com (H.S.F.); aalhajzen@hbku.edu.qa (A.A.H.Z.); maittaleb@hbku.edu.qa (M.A.); malkailani@hbku.edu.qa (M.I.A.); 2 Developmental Neurobiology Laboratory, Institute of Neuroscience, Université Catholique de Louvain, 1200 Brussels, Belgium; georges.chehade@uclouvain.be (G.C.); onchaii@hotmail.com (E.O.-C.L.)

**Keywords:** cell proliferation, cell migration, diaphanous-related formin 3, Dia2, Radmis

## Abstract

In humans, low expression of DIAPH3 is associated with poor prognosis in glioblastoma patients. In mice, DIAPH3 loss induces aberrant mitosis, chromosomal instability, and cell death; whereas the combined loss of DIAPH3 and p53 causes high-grade diffuse glioma and accelerates tumor onset. In this study, we used *Diaph3/p53* double conditional knockout mice (dcKO) and ultra-high-resolution MRI to detect and characterize the development and progression of glioma. We transcriptionally profiled early and advanced gliomas and identified stage-dependent alterations in molecular signatures as the tumor progresses. Notably, genetic pathways underlying self-renewal and extracellular matrix (ECM) interactions were upregulated at the expense of neural activity signature, which was severely shrunk. *CKAP2L* was identified as a differentially expressed gene at both early and advanced tumor stages in the dcKO mice. A subsequent siRNA screen to identify genes involved in proliferation and migration of glioblastoma cells identified *CKAP2L* as a top hit. Functional studies showed that *CKAP2L* depletion reduced proliferation and migration and induced cell accumulation in G_2_/M phase of the cell cycle. We also used data mining and thoroughly characterized the relationship between *CKAP2L* expression and glioma development. We found that *CKAP2L* expression correlates with proliferative and progenitor-associated markers, pointing to a potential role in maintaining highly proliferative and progenitor-like tumor cell state. We also identified a differentially methylated CpG site in the *CKAP2L* promoter that was associated with changes in gene expression across glioma datasets. Overall, our findings show that CKAP2L may contribute to glioma progression, help distinguish tumor grade, and represent a potential therapeutic target in glioblastoma treatment.

## 1. Introduction

Glioblastoma (GBM) is the most aggressive primary brain tumor in adults, characterized by rapid progression, extensive intratumoral heterogeneity and a median survival of approximately 15 months despite standard-of-care treatment. Current therapeutic strategies, including maximal surgical resection followed by radiotherapy and temozolomide, provide only limited survival benefit, likely due to the infiltrative nature of the tumor and the high rate of therapy resistance and recurrence [1]. At the cellular level, GBM is increasingly understood as a dynamic and heterogeneous microenvironment composed of distinct cellular states reminiscent of neural developmental lineages [2] where the progenitor-like glioma stem cells (GSCs) play a central role in tumor initiation and progression as well as recurrence due to their self-renewal capacities and therapy resistance [3]. Evidence from patient samples and mouse models shows that low-level driver mutations can exist in normal neural stem cells (NSCs) which may migrate and give rise to tumors elsewhere in the brain [4]. Glioma initiation is also linked to p53 loss combined with other oncogenic events which promotes expansion of progenitor-like cells [5]. Additionally, the disruption of mitotic regulation in neural progenitors leading to chromosomal instability and aberrant cell-cycle progression is one of the mechanisms underlying glioma genesis.

Diaphanous-related formin 3 (DIAPH3) is a cytoskeletal regulator involved in actin and microtubule dynamics which is specifically enriched in these neural progenitor cells [6]. Loss of DIAPH3 has been associated with mitotic defects and chromosomal instability, which can contribute to neoplastic transformation [7,8]. Interestingly, reduced DIAPH3 expression correlates with poor prognosis in GBM patients [9]. To better understand the mechanisms underlying glioma initiation, a genetically engineered mouse model was employed in which *Diaph3* and *Trp53* were conditionally deleted in neural progenitor cells and their progeny [6]. This model combined with the ultra-high-resolution MRI enables investigation of early tumorigenic events within controlled developmental and experimental settings. Notably, while Diaph3 loss alone was insufficient to induce tumor formation, its deletion in a *Trp53*-deficient background significantly increased the prevalence of high-grade diffuse gliomas and accelerated their onset [6]. These mice provide an in vivo experimental model to study how disruption of mitotic regulation in neural progenitors promotes glioma genesis and influences early molecular changes preceding and accompanying tumor development.

In this study, we investigated the molecular consequences of DIAPH3 loss using transcriptomic profiling of early and advanced tumor stages using samples from double conditional knockout mice. Through integrative analysis and functional screening, we identified CKAP2L as a key mediator of glioblastoma proliferation and migration. Furthermore, we explored its expression regulation across different glioma grades and showed its association with proliferative and progenitor-associated cellular features and tumor aggressiveness.

## 2. Materials and Methods

### 2.1. Analysis of CKAP2L Expression Across Databases

The developmental expression patterns of the genes of interest were assessed using transcriptomic data from the BrainSpan Atlas of the Developing Human Brain (www.brainspan.org). Normalized prenatal expression matrices and anatomical annotations were accessed directly through the BrainSpan online platform [10]. Three prenatal donors in the dataset were examined and exhibited highly similar spatial expression profiles. A representative donor was then selected for visualization.

Single-cell expression profiles were examined using a publicly available single-cell RNA-sequencing dataset of human glioblastoma (SCP393), accessible through the Broad Institute Single Cell Portal (https://singlecell.broadinstitute.org). This dataset includes malignant and non-malignant cell populations profiled from patient tumors, annotated into established transcriptional states, including neural-progenitor-like (NPC-like), oligodendrocyte-progenitor-like (OPC-like), mesenchymal-like (MES-like) and astrocyte-like (AC-like) clusters.

### 2.2. High-Content siRNA Screening for Proliferation and Migration

To identify the role of differentially expressed gene hits in cell migration and proliferation, we have conducted a high-content phenotypic siRNA screen of pre-designed ON-TARGETplus™ SMARTpool siRNA library (Horizon Discovery, Dharmacon, CO, USA) targeting the selected 85 genes in U251 CTL and U251 DIAPH3-KO cells (Supplementary Table S1). The siRNA screen was performed in technical triplicate in 96-well plates. Each plate included a non-targeting control siRNA (NTC) as negative controls (*n* = 10) and PLK1 siRNA as a positive control (*n* = 2). PLK1 was used as positive functional control to assess the siRNA transfection efficacy. The siRNA transfection steps and media change were performed using an automated liquid handling mode of VIAFLO 96 system (INTEGRA Biosciences AG, Zizers, Switzerland). Cells (5000 cells per well) were reverse transfected with siRNA with a final concentration of 24 nM using Lipofectamine™ RNAiMAX (Invitrogen, Thermo Fisher Scientific, CA, USA, 13778075). The culture medium was replaced with fresh complete media 24 h after transfection. The plates were then incubated for an additional 48 h until a confluent cell monolayer was observed in the control wells. Next, scratch wounds were created automatically across the siRNA-transfected cell monolayer wells using the BioTek AutoScratch wound making tool (Agilent Technologies (BioTek), Winooski, VT, USA). The plates were then washed and a pre-migration bright-field image (t0) was taken immediately after the cell scratch using the Operetta CLS High-Content Imaging System (PerkinElmer, Hamburg, Germany). Then, the cells were allowed to migrate into the clear detection zone for 24 h (t24). Cells were then fixed with PFA 4% and stained with green cell mask (Invitrogen, Thermo Fisher Scientific, Carlsbad, CA, USA) and DAPI (Invitrogen, Thermo Fisher Scientific, Carlsbad, CA, USA). Next, the stained plates were imaged at 10× magnification using the Operetta CLS High-Content Imaging System. The nine imaged fields of view were then stitched together for subsequent image analysis. The gap areas were quantified using the Harmony^®^ image analysis software (PerkinElmer, Hamburg, Germany). The effect of siRNA knockdown on cell migration was assessed by calculating the gap closure (%) using the following formula:

Gap closure %=(1−Gap area t24Gap area t0)×100

To assess cell proliferation, nuclei segmentation and automated cell counting were performed using the Harmony^®^ analysis software. All extracted migration and cell count values of treated wells were normalized to the non-targeting control (NTC) mean for each plate. Then, the normalized values of technical replicate plates were averaged.

### 2.3. Spheroid Formation and Radial Migration Assay

U251 CTL and *CKAP2L* KO cells were seeded into low-attachment 12-well plates (pre-coated with 1% agarose) at a density of 15,000 cells per well to initiate spheroid formation. Spheroids were maintained for 7 days with half-medium change every 48 h. Spheroid morphology was monitored every 24 h to assess the size and structural organization. Fiji software (Fiji Is Just ImageJ, ImageJ 2.15.0) was used for spheroids size quantification. For the radial migration assay, spheroids were stained with Hoechst 33342 (Invitrogen, Thermo Fisher Scientific, Carlsbad, CA, USA) at a final concentration of 5 µg/mL for 10 min at 37 °C. Spheroids were transferred into standard 12-well plates containing fresh complete medium to allow for attachment and radial outgrowth. Images of spheroid spreading were captured every 24 h under adherent conditions. The Hoechst-stained images were then converted to an 8-bit grayscale in ImageJ and then analyzed using the Spheroid Invasion Analysis Python (version 3.15.2) pipeline available at: https://github.com/rogerh2/SpheroidInvasionAnalysis (accessed on 17 August 2026) [11].

Detailed experimental protocols and extended descriptions of bioinformatics and statistical analyses are provided in the Supplementary Materials.

## 3. Results

### 3.1. Transcriptomic Profiling of Gliomas

**Figure 1:**
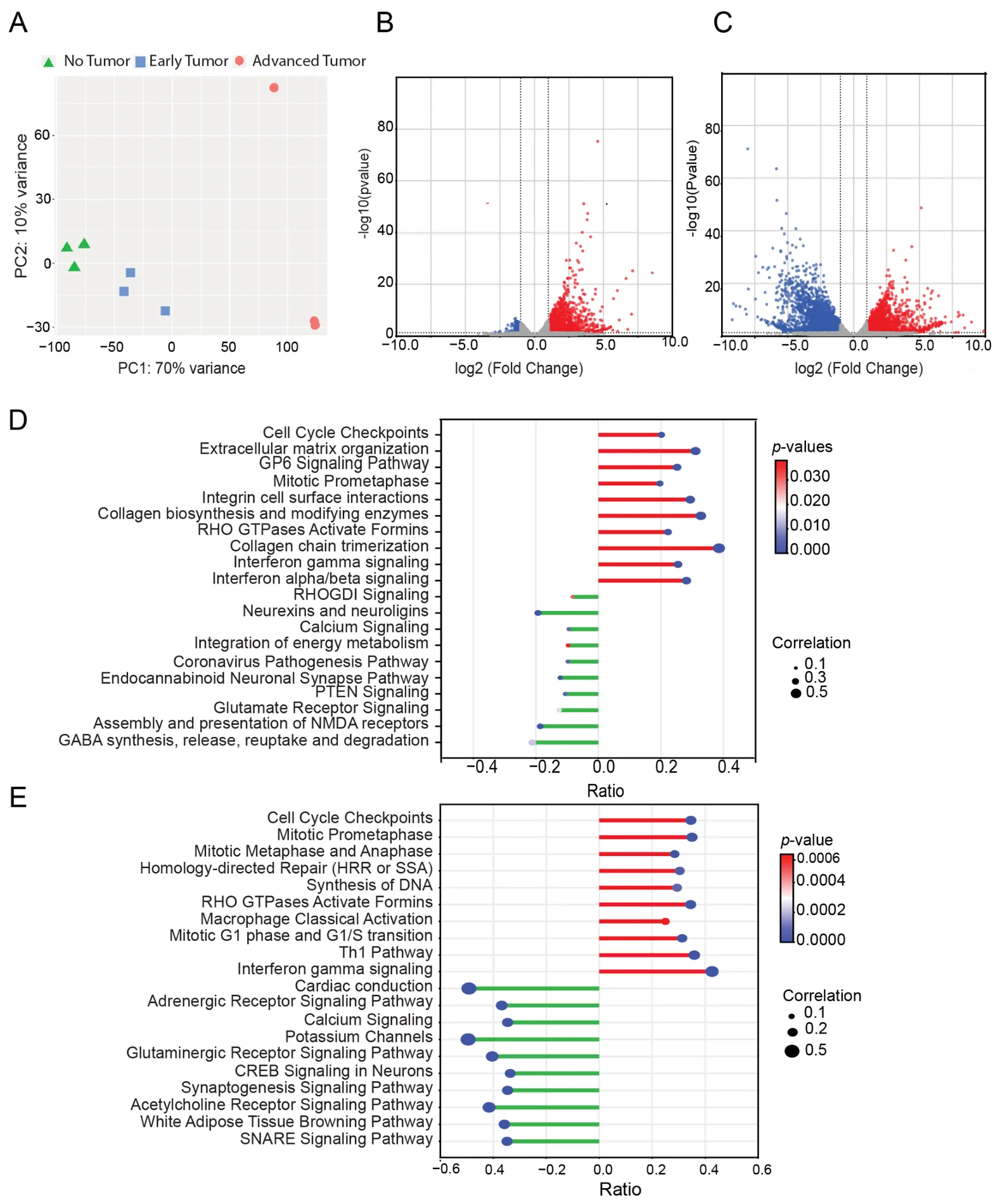
Differential gene expression analysis of early- and advanced-stage tumors. (**A**) PCA for transcriptomic separation between early tumors, advanced tumors, and control brain samples. (**B**) Volcano plot for differentially expressed genes between early tumor and control groups (|log_2_FC| ≥ 1, adj. *p* < 0.05). (**C**) Volcano plot displaying DEGs between advanced and early tumor samples (|log_2_FC| ≥ 1, adj. *p* < 0.05). (**D**,**E**) Pathway enrichment from IPA illustrating activated signaling pathways (red lines) and inhibited signaling pathways (green lines) in early-stage tumors (**D**) and advanced-stage tumors (**E**).

To investigate the molecular alterations underlying tumor development, we conducted an RNA-seq analysis of gliomas derived from cortex-specific double conditional knockout mice (*Diaph3*/*Trp53 dcKO*) [6]. Principal component analysis (PCA) showed a clear separation between control, early tumor and advanced tumor samples (*n* = 3 per genotype), indicating distinct transcriptional profiles (Figure 1A). Advanced tumor samples clustered separately from both control and early tumor samples. A differential gene expression analysis was performed to quantify inter-stage transcriptional differences. Prior to analysis, pseudogenes and long non-coding RNAs (lncRNAs) were excluded, and only protein-coding genes were retained. Comparison of early olfactory bulb tumors with matched non-tumor controls (dcKO tissue from the contralateral olfactory bulb), identified 1210 differentially expressed genes (DEGs) including 987 upregulated and 223 downregulated genes (Figure 1B, Supplementary Table S2). Comparison of advanced versus early tumors identified 3727 DEGs with 1488 upregulated and 2237 downregulated genes (Figure 1C, Supplementary Table S3). Pathway enrichment analysis was then conducted on DEGs across tumor stages. In early tumors compared to non-tumor controls (Figure 1D, Supplementary Table S4), upregulated pathways were primarily associated with cell cycles, including cell cycle checkpoints and mitotic signaling as well as Rho GTPase signaling and Rho GTPase-activated formins. Extracellular matrix-related pathways, including collagen and integrin signaling, were also enriched among upregulated genes. In contrast, downregulated pathways were predominantly related to neuronal and synaptic functions (Figure 1D). In advanced tumors compared to early-stage tumors (Figure 1E, Supplementary Table S5), upregulated pathways pointed to cell cycle and mitotic processes along with DNA replication and DNA repair pathways. Rho GTPase signaling remained enriched and additional enrichment of immune- and inflammation-related pathways were observed. Downregulated pathways were consistently associated with neuronal and neurogenic processes such as synaptic signaling and neuronal differentiation (Figure 1E). A Gene Ontology (GO) enrichment analysis was performed as a complementary approach to characterize the functional categories associated with DEGs. At the early stage, upregulated genes were enriched in cell cycle-related processes across biological processes, cellular components and molecular functions. Extracellular matrix organization-related terms were also identified (Supplementary Figure S1A). Downregulated genes were primarily enriched in neuronal and synaptic activity-related categories (Supplementary Figure S1B). At the advanced stage, upregulated genes remained enriched in cell cycle and mitotic processes with additional enrichment of immune- and inflammation-related categories (Supplementary Figure S2A). Downregulated genes highlighted neuronal and neurogenic terms (Supplementary Figure S2B).

### 3.2. Stage-Dependent Transcriptional Reprogramming During Tumor Progression

**Figure 2:**
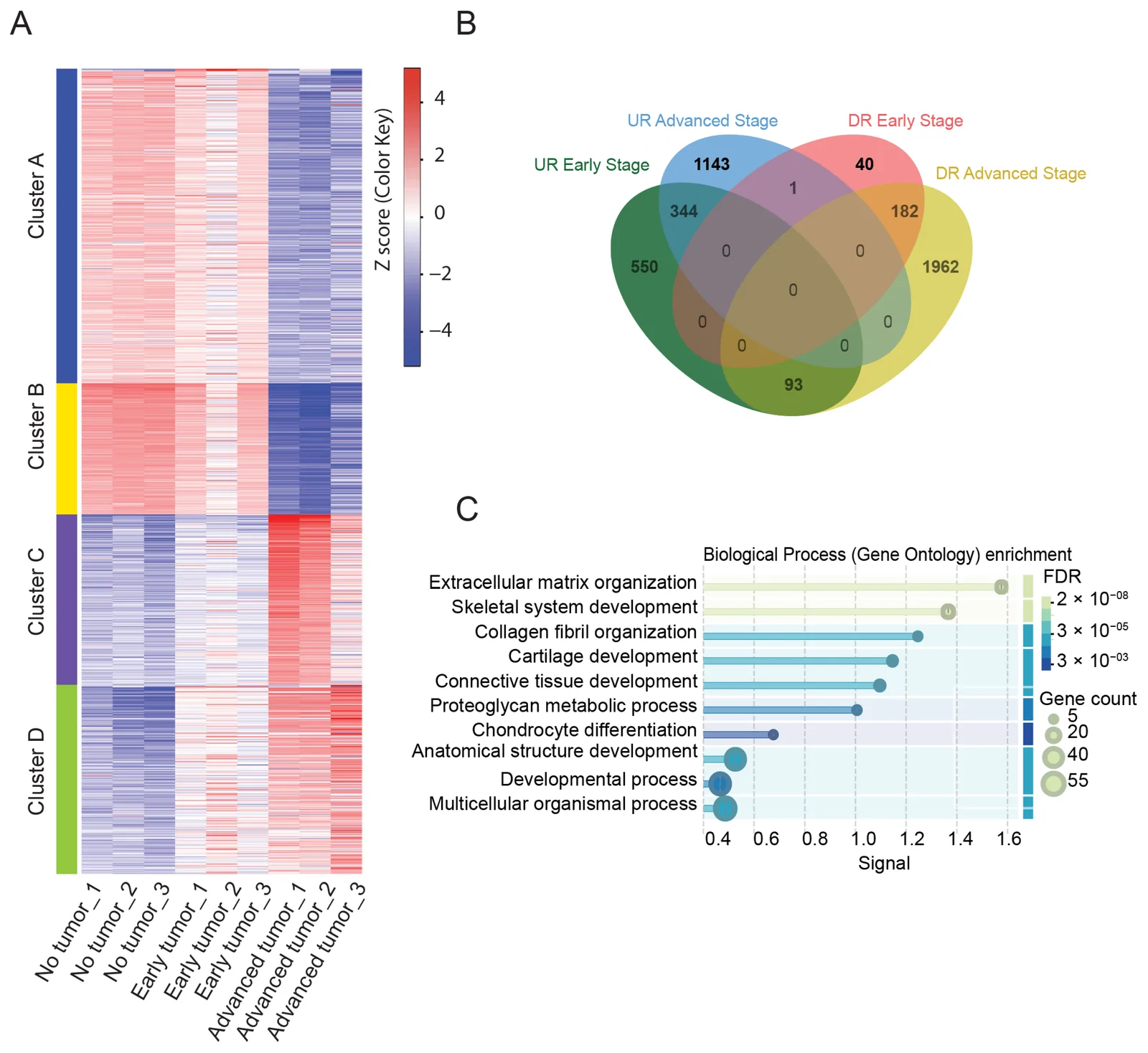
Clustering and functional characterization of gene expression dynamics during tumor progression. (**A**) K-means clustering of DEGs according to their expression in control tissue (No tumor_1–3), early-stage tumors (Early Tumor_1–3) and advanced-stage tumors (Advanced tumor_1–3), revealing four major expression clusters with distinct temporal patterns. Clustering was performed using the iDEP platform (k = 4) which applies internal normalization and distance-based statistical partitioning to group genes with similar expression trajectories. (**B**) Venn diagram showing the overlap between upregulated (UR) and downregulated (DR) genes at early versus advanced tumor stages. The diagram highlights stage-specific transcriptional programs as well as shared DEGs across tumor progression. (**C**) GO enrichment analysis of the 93-gene cluster characterized by upregulation in early-stage tumors and downregulation in advanced-stage tumors. FDR: False Discovery Rate.

To assess gene expression dynamics as the tumors progress, K-means clustering was applied to analyze the transcriptomic data. Genes were grouped into four distinct clusters based on their expression profiles (Figure 2A, Supplementary Table S6). Clusters A and B comprised genes highly expressed in control samples and downregulated in tumors. They were enriched for ion transport, voltage-gated channel activity, and synaptic signaling. In contrast, Cluster C included genes progressively upregulated from early to advanced stages and associated with immune-related functions such as cytokine signaling, chemokine binding and antigen receptor activity. Cluster D included genes enriched for cell cycle-related processes including DNA replication, helicase activity and microtubule-based functions.

To identify unique versus shared gene signatures, we used an overlap analysis and identified stage-specific and common gene sets (Figure 2B). Notably, SV2C (Synaptic Vesicle Glycoprotein 2C) shifted from downregulation in early tumors to upregulation in advanced tumors, while 93 genes showed the opposite pattern being upregulated at the early stage and downregulated at the advanced stage. Gene Ontology analysis of these 93 genes revealed enrichment in extracellular matrix organization and cellular component assembly (Figure 2C).

### 3.3. High-Content siRNA Screening of Selected DEGs Identified Hits Associated with Proliferation and Migration

**Figure 3:**
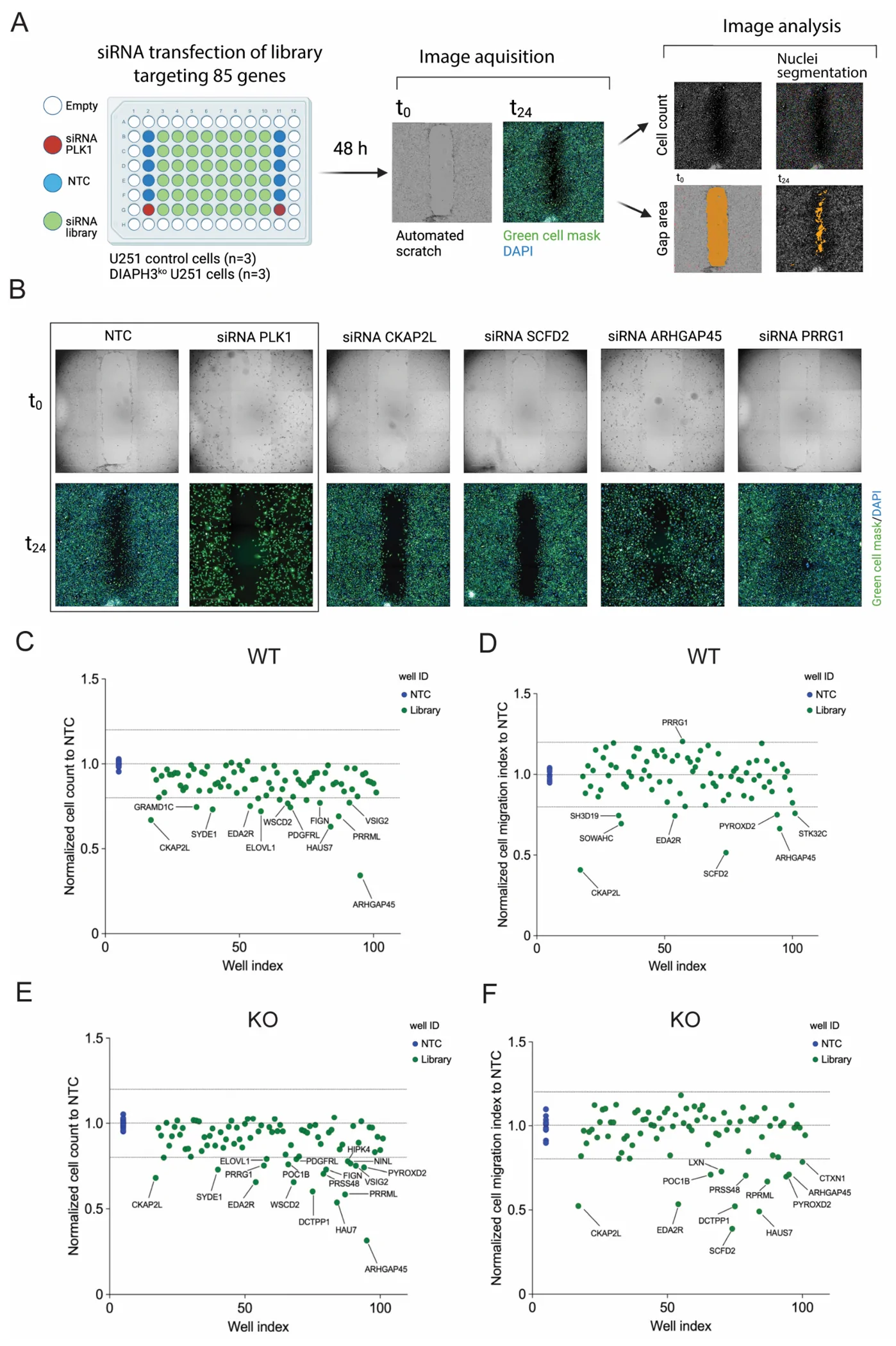
High-content siRNA screen assessing proliferation and migration in U251 and U251-DIAPH3 KO glioblastoma cells. (**A**) Workflow of siRNA screen using the scratch migration assay. (**B**) Representative images of U251 (CTL) cells from the siRNA screen showing control wells (non-targeting control (NTC) and siRNA PLK1), and selected siRNAs that altered cell migration. (**C**–**F**) Distribution of normalized cell count and migration indices following siRNA-mediated knockdown of 85 candidate genes in U251 WT and DIAPH3-KO cells. Horizontal dashed lines indicate the selection thresholds used to identify candidate hits. Values of cell count in WT cells (**C**) and in DIAPH3-KO cells (**E**) and migration index in WT cells (**D**) and in DIAPH3-KO cells (**F**) were normalized to NTC. Blue dots represent NTC replicates, and green dots represent the siRNAs library. Dashed lines indicate ± 4 × SD thresholds from NTC.

To functionally assess candidate genes identified from transcriptomic analyses, a high-content screen was performed in U251 cells (CTL) and DIAPH3 KO U251 cells using a siRNA library targeting 85 DEGs that have not been extensively investigated in glioma development (Figure 3A). The screen yielded a Z’-factor of 0.82 ± 0.07, falling well within the excellent range (>0.5) and confirming the robustness and sensitivity of the assay. siRNA hits were identified by applying a cut-off value corresponding to the NTC normalized mean ± 4 × SD. The knockdown of most DEGs showed minimal effects and clustered near baseline levels for both proliferative and migration activity (Figure 3B, Supplementary Table S7). Eight siRNAs inhibited migration, and 15 siRNAs inhibited proliferation in U251 control cells (Figure 3C,D). In contrast, 12 siRNAs inhibited migration, and 19 siRNAs inhibited proliferation in DIAPH3 KO U251 cells (Figure 3E,F). Scatter plots of normalized migration versus proliferation relative to the NTC-highlighted genes were associated with reduced proliferation, migration, or both (Supplementary Figure S3A,B). To prioritize candidates for further analysis, DEGs were ranked based on their effects on proliferation and migration (Supplementary Figure S3C,D). Among the tested genes, *CKAP2L* consistently showed a strong effect on cell proliferation and migration and was therefore selected for further validation and functional analysis. *CKAP2L* was identified in the RNA-seq dataset from the *Trp53/Diaph3* dcKO mouse model as differentially expressed at both early and advanced tumor stages, with log_2_ fold changes of 2.91 (*p* = 4.05 × 10^−10^) and 2.56 (*p* = 1.85 × 10^−13^), respectively (Supplementary Tables S2 and S3). Integration of the transcriptomic data with an Ingenuity Pathway Analysis (IPA) further identified CKAP2L among the top predicted regulators associated with glioma genesis and progression (Supplementary Table S8). An IPA network centered on CKAP2L revealed interactions with genes involved in cell proliferation, cell viability and cell death (Supplementary Figure S4A). The Gene Ontology analysis of these associated genes demonstrated significant enrichment in processes related to the cell cycle and cell division, including cell cycle progression and chromosome segregation (Supplementary Figure S4B).

### 3.4. CKAP2L Expression and Patient Survival in Gliomas

**Figure 4:**
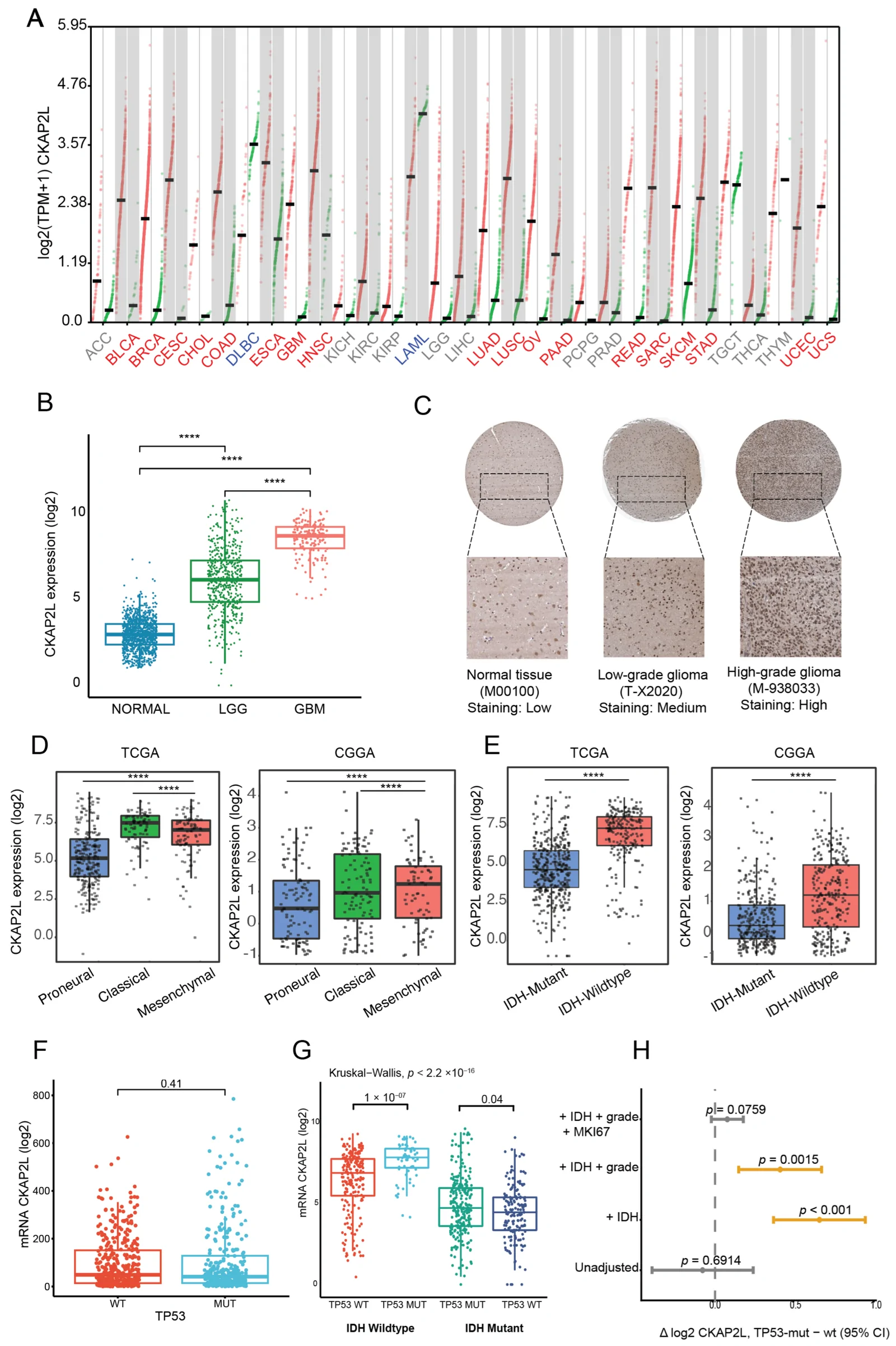
*CKAP2L* as a marker for high-grade and more aggressive glioma subtypes. (**A**) *CKAP2L* expression levels in diverse types of cancer and corresponding normal tissues from the TCGA project using GEPIA (https://gepia3.bioinfoliu.com/). Red: cancer, green: normal tissue. (**B**) Boxplot showing *CKAP2L* mRNA expression levels in normal brain tissues, low-grade glioma (LGG) and glioblastoma (GBM) samples from TCGA. (**C**) Immunohistochemistry (HPA039407 antibodies) images from the Human Protein Atlas database showing CKAP2L protein levels in normal brain tissue, LGG, and high-grade glioma. (**D**,**E**) *CKAP2L* expression distribution in the TCGA and CGGA datasets according to molecular subtype (**D**) and IDH status (**E**). (**F**) *CKAP2L* mRNA expression in the *TP53*-wild-type (WT) and *TP53*-mutant (MUT) gliomas. The difference between groups was not statistically significant (*p* = 0.41). (**G**) Expression of *CKAP2L* according to IDH and *TP53* status. (**H**) Linear regression of the differences in *CKAP2L* expression between *TP53*-mutant and wild-type tumors in unadjusted and progressively adjusted models for IDH status; IDH status and tumor grade; and IDH status, tumor grade, and *MKI67* expression. ACC: adrenocortical carcinoma, BLCA: bladder urothelial carcinoma, BRCA: breast invasive carcinoma, CESC: cervical squamous cell carcinoma and endocervical adenocarcinoma, CHOL: cholangiocarcinoma, COAD: colon adenocarcinoma, DLBC: diffuse large B-cell lymphoma, ESCA: esophageal carcinoma, GBM: glioblastoma multiforme, HNSC: head and neck squamous cell carcinoma, KICH: kidney chromophobe, KIRC: kidney renal clear cell carcinoma, KIRP: kidney renal papillary cell carcinoma, LAML: acute myeloid leukemia, LGG: brain lower-grade glioma, LIHC: liver hepatocellular carcinoma, LUAD: lung adenocarcinoma, LUSC: lung squamous cell carcinoma, OV: ovarian S cystadenocarcinoma, PAAD: pancreatic adenocarcinoma, PCPG: pheochromocytoma and paraganglioma, PRAD: prostate adenocarcinoma, READ: rectum adenocarcinoma, SARC: sarcoma, SKCM: skin cutaneous melanoma, STAD: stomach adenocarcinoma, TGCT: testicular germ cell tumors, THCA: thyroid carcinoma, THYM: thymoma, UCEC: uterine corpus endometrial carcinoma, UCS: uterine carcinosarcoma, TPM: transcripts per million. Statistical significance is indicated as **** *p* < 0.0001.

To further characterize *CKAP2L*, its expression and associated transcriptional features were examined using various datasets. Expression analysis in multiple cancers from the TCGA project [12] and the corresponding normal tissue from the Genotype-Tissue Expression (GTEx) project [13] revealed high expression of *CKAP2l* in 18 tumor types with markedly elevated levels in glioblastoma (Figure 4A). In gliomas, *CKAP2L* expression showed a progressive increase from normal brain tissues (GTEx) to lower-grade glioma (LGG) and was the highest in GBM (Figure 4B). This expression pattern was confirmed at the protein level by immunohistochemical staining (Figure 4C). Stratification by molecular subtype showed higher *CKAP2L* expression in classical and mesenchymal gliomas compared to proneural gliomas in both TCGA and CGGA cohorts (Figure 4D). Stratification by IDH status showed that IDH-wild-type tumors expressed higher levels of *CKAP2L* compared to IDH-mutant tumors (Figure 4E). Stratification by TP53 mutation status did not reveal any significant change in *CKAP2L* expression between TP53-mutant and TP53-wild-type tumors (*p* = 0.41, Figure 4F). Stratification by *TP53* and IDH status together showed that TP53-mutant tumors expressed higher levels of *CKAP2L* than *TP53*-wild-type-tumors, with a greater difference in IDH-wild-type gliomas (*p* = 1 × 10^−7^, Figure 4G) compared to IDH-mutant gliomas (*p* = 0.04, Figure 4G). Linear regression analysis showed that the association between *TP53* mutation status and *CKAP2L* expression became significant following adjustment for IDH (*p* < 0.001), IDH and WHO grade (*p* = 0.0015), but not following additional adjustment for *MKI67* expression (*p* = 0.0759) (Figure 4H).

We evaluated the prognostic value of *CKAP2L* expression in relation to patient clinical outcomes next. The Kaplan–Meier survival analysis revealed that elevated *CKAP2L* expression was significantly associated with poorer overall survival in the combined TCGA glioma cohort (HR = 5.43, 95% CI: 3.78–7.80; *p* < 0.0001; Supplementary Figure S5A). However, this association was not preserved within the GBM cohort alone (HR = 0.77, 95% CI: 0.49–0.19; *p* = 0.22; Supplementary Figure S5B), suggesting that the survival differences observed in the combined cohort likely reflect disparities between the LGG and GBM patients rather than *CKAP2L* expression. The Univariate Cox regression analysis revealed that *CKAP2L* expression was significantly associated with overall survival (*p* < 0.001; Supplementary Figure S5C). However, this association lost significance in the multivariate model after adjusting for WHO grade, MGMT promoter methylation status, age, sex, IDH mutation status, and 1p/19q co-deletion (*p* = 0.139; Supplementary Figure S5D). A stratified multivariable analysis showed that, while *CKAP2L* expression was not independently associated with survival in IDH-wild-type gliomas (*p* = 0.601; Supplementary Figure S5E), higher expression was significantly associated with increased mortality in the IDH-mutant subgroup (HR = 1.91, 95% CI: 1.05–3.48; *p* = 0.034; Supplementary Figure S5F). We tested the *CKAP2L-*IDH interaction and found that it was not statistically significant (likelihood-ratio test, χ^2^ = 2.13, df = 1, *p* = 0.145). Therefore, the observed differences should be interpreted cautiously and considered as exploratory. Taken together, these results imply that *CKAP2L* may not serve as a reliable independent prognostic marker.

To explore potential regulatory mechanisms underlying *CKAP2L* expression, DNA methylation analysis was performed using the GBM and LGG cohorts accessed through the UCSC Xena Browser [14]. Examination of the *CKAP2L* promoter region identified ten CpG sites profiled across samples (Supplementary Figure S6A). Among these, one CpG site (cg00908571) displayed a distinct DNA methylation profile (Supplementary Figure S6B). Further analysis of this CpG site revealed a clear grade-dependent trend, with significantly higher methylation levels in LGG and progressive hypomethylation in GBM samples (Supplementary Figure S6C). The decrease in methylation coincided with increased *CKAP2L* expression. To further characterize this relationship, a Spearman correlation analysis was performed across the overall cohort and stratified by IDH status. A significant inverse correlation was observed in the overall cohort (*n* = 507; ρ = −0.45, *p* < 0.001 Supplementary Figure S6D), IDH-mutant gliomas (*n* = 377; ρ = −0.20, *p* < 0.001 Supplementary Figure S6D), and IDH-wild-type gliomas (*n* = 130; ρ = −0.59, *p* < 0.001 Supplementary Figure S6D), with a stronger association in IDH-wild-type tumors. These findings support an inverse association between cg00908571 methylation and *CKAP2L* expression before and after IDH status stratification.

### 3.5. CKAP2L Expression Is Associated with Proliferative and Progenitor-like Cellular State in Glioblastoma

**Figure 5:**
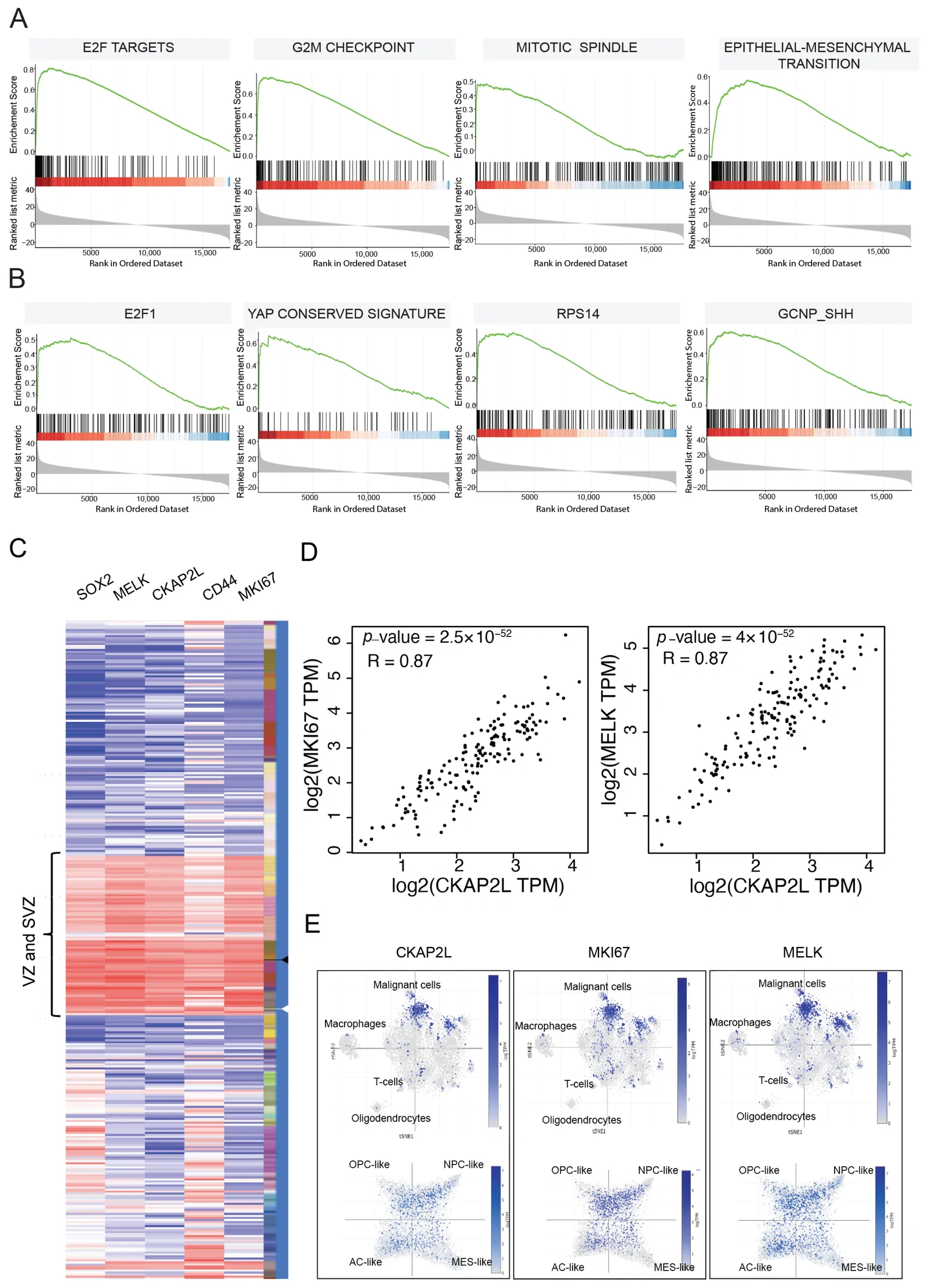
*CKAP2L* is associated with neural progenitor-like states in developing human brains and glioblastomas. (**A**,**B**) Gene set enrichment analysis results showing plots for hallmarks: mitotic spindle (NES = 2.16, FDR q = 0.001), G2/M checkpoint (NES = 1.81, FDR q = 0.082), E2F targets (NES = 1.70, FDR q = 0.107) epithelial–mesenchymal transition (EMT; NES = 1.53, FDR q = 0.147) (**A**) and oncogenic pathways: E2F1_(NES = 1.95, FDR q = 0.007), YAP_CONSERVED_SIGNATURE (NES = 1.77, FDR q = 0.009) RPS14 (NES = 1.87, FDR q = 0.005) and GCNP_SHH_UP_LATE (NES = 1.92, FDR q = 0.004) (**B**). (**C**) Representative heatmap of *CKAP2L* and neural progenitor markers expression (microarray data) from the BrainSpan Atlas. (**D**) Correlation analysis of TCGA GBM tumors using GEPIA demonstrates a strong positive association between *CKAP2L* and *MKI67* and *MELK* mRNA levels. (**E**) Single-cell RNA-seq data from GBM patient samples show overlapping expression patterns of *CKAP2L*, *MKI67* and *MELK*. YAP: Yes-Associated Protein, RPS14: Ribosomal Protein S14 Pathway, GCNP_SHH: Granule Cell Neuron Precursor—Sonic Hedgehog Signaling, SZ: subventricular zone, VZ: ventricular zone.

We performed a gene set enrichment analysis (GSEA) [15] on the TCGA GBM/LGG cohort by stratifying patients into *CKAP2L-*high and *CKAP2L*-low expression groups. A hallmark enrichment analysis revealed that tumors with high *CKAP2L* expression were significantly enriched for cell cycle-related processes, including the G_2_/M checkpoint, mitotic spindle organization, and E2F target gene sets, as well as the epithelial–mesenchymal transition (EMT), indicating association with proliferative and invasive tumor signals (Figure 5A). An analysis of oncogenic signatures further showed enrichment of multiple pathways in *CKAP2L*-high tumors (Figure 5B), including E2F1, YAP1_CONSERVED_SIGNATURE (Hippo/YAP signaling associated with proliferation and stemness), SHH (Sonic Hedgehog signaling, linked to developmental and stem-like programs), and RPS14 (ribosomal protein-associated signature linked to cellular growth and proliferation).

To investigate whether *CKAP2L* expression is associated with a progenitor-like and proliferative cellular context, we first examined its expression pattern in the developing human brain using the BrainSpan dataset. (Figure 5C). *CKAP2L* was predominantly expressed in the ventricular and subventricular zones, regions enriched in neural progenitor populations, where its expression spatially overlapped with progenitor-associated markers, including *SOX2* and *CD44*, as well as the proliferation-associated markers *MKI67* and *MELK* (Figure 5C). We examined next whether this relationship was also observed in human glioblastoma. In the TCGA-GBM samples, *CKAP2L* expression positively correlated with *MKI67* and *MELK* expression (Figure 5D). Finally, an analysis of single-cell RNA-sequencing data showed that *CKAP2L*, MKI67, and *MELK* were co-expressed within malignant cell populations (Figure 5E). Thus, across the developmental brain tissue and GBM samples, *CKAP2L* expression was consistently associated with proliferative cellular populations.

### 3.6. Loss of CKAP2L Impairs Proliferation, Migration, and Cell-Cycle Progression

**Figure 6:**
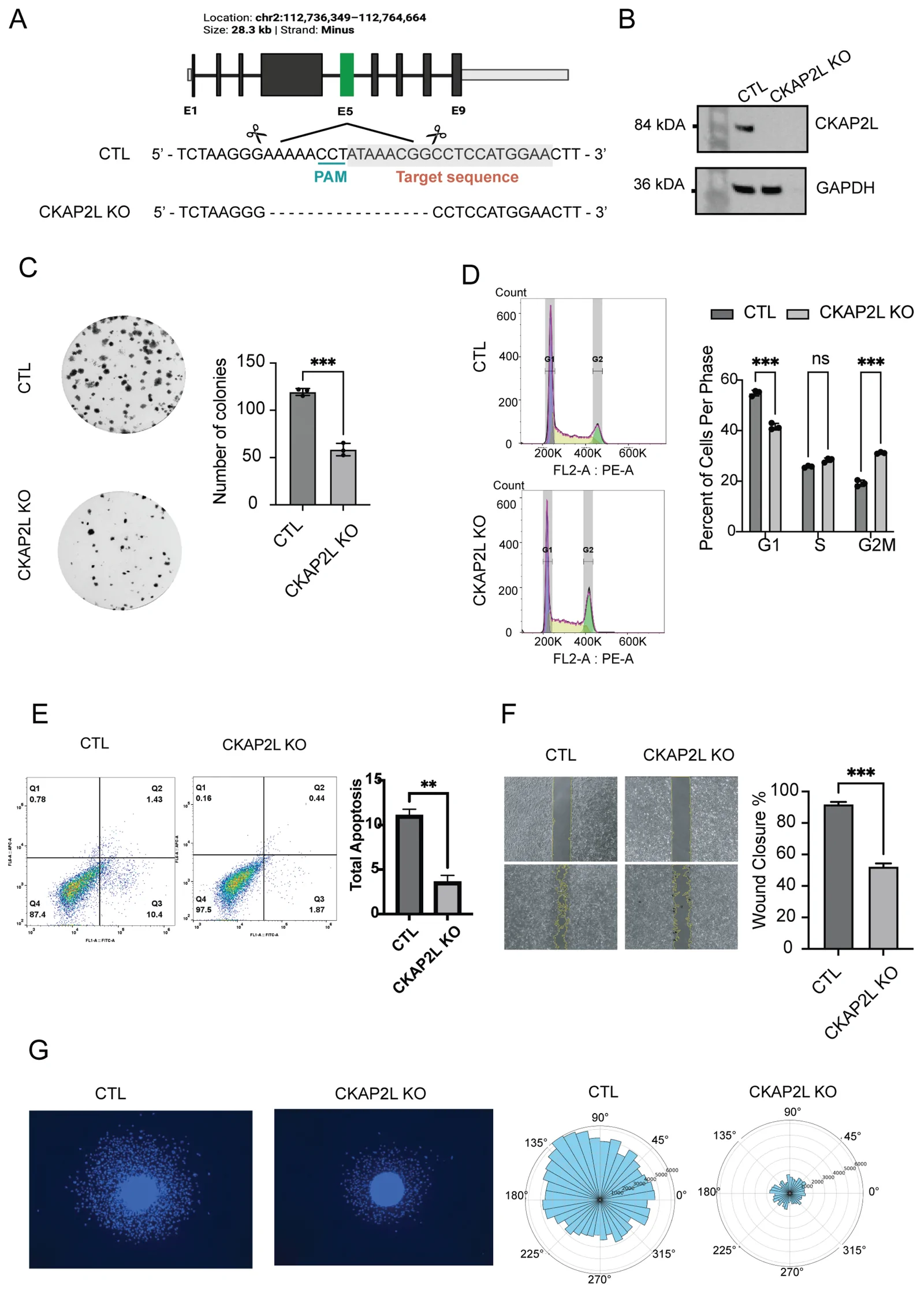
*CKAP2L* loss impairs cell cycle, proliferation, and migration in GBM cells in vitro. (**A**) Schematic representation of the *CKAP2L* genomic locus indicating the CRISPR–Cas9 target site. The guide RNA is in exon 5, and the protospacer adjacent motif (PAM) sequence is indicated. Deletion of 16-nucleotides in *CKAP2L* knockout (KO) cells was confirmed by Sanger sequencing. (**B**) Western blot analysis of *CKAP2L* protein in *CKAP2L* KO cells compared with control (CTL) cells. (**C**) Colony formation assay with quantification in *CKAP2L* KO cells compared with CTL cells. (**D**) Cell-cycle analysis by flow cytometry showing the distribution of cells across G_1_, S, and G_2_/M phases in CTL and *CKAP2L* KO cells. (**E**) Flow cytometry analysis of apoptosis in control and KO cells. Cells were divided into four quadrants representing viable (Q4), early apoptotic (Q3), late apoptotic (Q2), and necrotic (Q1) populations. (**F**) Wound healing assay in *CKAP2L* KO cells compared with CTL cells. (**G**) Radial migration assay showing the spatial distribution and directional migration of Hoechst-stained cells. Statistical significance is indicated as ** *p* < 0.01, *** *p* < 0.001, and ns = not significant.

To assess the role of *CKAP2L*, we used CRISPR/Cas9 in U251 cells to generate a *CKAP2L* knockout (KO) cell line. Gene editing was confirmed by sequencing, which identified a 16-nucleotide deletion at the target site (Figure 6A). This frameshift mutation introduced a premature stop codon and presumably nonsense-mediated mRNA decay. Western blot analysis confirmed the loss of *CKAP2L* expression (Figure 6B, Supplemental File 1). *CKAP2L* KO exhibited a reduction in both colony number and size compared to control cells (Figure 6C). Cell-cycle analysis revealed a decrease in the G_1_ population and an accumulation of cells in the G_2_/M phase, while the S phase fraction remained unchanged (Figure 6D). Apoptosis was assessed to determine whether reduced cell growth was associated with increased cell death. *CKAP2L* KO showed a significant reduction in the number of apoptotic cells compared with control cells (Figure 6E), indicating that apoptosis cannot account for the reduction in colony numbers in *CKAP2L* KO cells.

Cell migration was evaluated using wound healing assays under low-serum conditions. *CKAP2L* KO displayed reduced wound closure compared to control cells (Figure 6F). Three-dimensional culture assays were performed using low-attachment conditions to assess spheroid formation and outgrowth. Control cells formed compact, spherical structures, whereas *CKAP2L* KO formed less organized and irregular structures. Upon re-plating, *CKAP2L* KO exhibited reduced radial outgrowth compared to control cells (Figure 6G).

## 4. Discussion

In this study, we investigated the molecular changes associated with glioma development and progression in a *Diaph3/Trp53* double-knockout model. A transcriptomic analysis of early tumors revealed enrichments in a) mitotic signaling, suggesting active tumor cell proliferation, which is consistent with our previous findings that the DIAPH3 loss induces aneuploidy and mitotic catastrophe [7,8]. These defects, combined with loss of p53, which impairs surveillance mechanisms [16], allow defective cells to evade elimination and undergo uncontrolled proliferation [17]. b) Rho GTPase signaling, which functions upstream of DIAPH3 [18], was accompanied with the downregulation of negative regulators of Rho GTPases [19]. Given the involvement of Rho GTPase signaling in multiple oncogenic processes [20], its modulation may be relevant to the earlier tumor onset observed in *Diaph3/Trp53* double-knockout mice [6]. c) Enrichment of the ECM-related pathways suggests early activation of tumor–microenvironment interactions that promote cell growth and survival [21]. As tumors advanced, immune and inflammatory pathways became more enriched, which is consistent with previous studies showing a transition from active inflammation to immune exhaustion during glioblastoma progression [22]. The observed enhancement of cell cycle and DNA repair signaling as the tumor progresses suggests that tumor cells maintain proliferative capacity despite genomic instability caused by the DIAPH3 loss. In contrast, we observed a marked loss of neuronal identity characterized by a downregulation of neuronal gene expression. This observation is consistent with previous studies showing reduced neuronal signaling pathways in human glioblastoma [23]. The decline in neuronal lineage signaling may reflect tumor dedifferentiation, as loss of neuronal markers has been associated with more aggressive GBM subtypes and poorer prognosis [24,25].

According to our siRNA screening, DIAPH3 loss affects the cellular response to gene silencing in that a greater number of siRNAs altered proliferation and migration in *DIAPH3* knockout cells compared to control cells. The absence of DIAPH3 may increase cellular vulnerability to perturbations by impairing actin and microtubule dynamics required for faithful mitosis [7,8]. This is consistent with in vivo findings that showed that the *Trp53/Diaph3* double-knockout mouse model developed tumors earlier than the *Trp53* single knockout. The loss of DIAPH3 in neural stem cells could increase their susceptibility to external stressors, including epigenetic alterations or accumulation of secondary mutations, predisposing them to malignant transformation. It also aligns with human GBM data which shows that low *DIAPH3* expression correlates with poor prognosis.

The enhanced expression of *CKAP2L* throughout tumor progression, together with its inhibitory effects on proliferation and migration upon downregulation, supports a role for *CKAP2L* in sustaining glioma growth and invasiveness. Accordingly, analysis of human GBM single-cell data showed that *CKAP2L* is expressed specifically in highly proliferative, progenitor-like cells, along with *MKI67* and *MELK* [26,27]. In the combined glioma cohort, *CKAP2L* expression did not differ significantly according to TP53 mutation status; however, stratified analyses revealed higher expression in *TP53*-mutant tumors, particularly in the IDH-wild-type gliomas. The progressive increase in *CKAP2L* expression with glioma grade, together with its enrichment in classical, mesenchymal, and IDH-wild-type tumors, supports a role for CKAP2L in driving aggressive and therapy-resistant glioma states [28,29]. Regarding clinical outcome, the apparent association between high *CKAP2L* expression and overall survival in the combined glioma cohort may reflect a tumor grade and molecular subtype rather than an independent prognostic effect.

The enrichment of E2F, G2/M checkpoint and mitotic spindle signatures is consistent with its established functions in mitotic progression and chromosome segregation [30,31]. High *CKAP2L* expression was consistently associated with oncogenic programs such as E2F1, YAP and SHH signaling known to be implicated in the tumorigenic capacity in glioblastoma [32,33,34]. The TCGA-LGG/GBM classification used in this study was based on a histological classification which does not fully reflect the current molecular WHO classification, and further validation in recent clinical cohorts is needed.

CKAP2L deletion markedly impaired colony-forming capacity, supporting the contribution of CKAP2L in maintaining the proliferative and clonogenic properties of glioblastoma cells required for sustained tumor cell growth. Cell-cycle analysis revealed a redistribution of cells following CKAP2L deletion, characterized by a decrease in G_1_ and accumulation in G_2_/M phase. The cell death analysis revealed less apoptotic cells in *CKAP2L* knockout cells compared to control. These observations suggest that the growth defects induced by CKAP2L deletion are not primarily mediated through apoptosis but rather through altered cell cycle dynamics. *CKAP2L* knockout cells displayed a reduced migratory behavior in wound-healing assays, a finding that was further validated using radial migration assays from the 3D spheroid outgrowth. Thus, CKAP2L may contribute to tumor cell motility and invasive potential, in agreement with its role in microtubule-associated processes [30,31]. These findings require further validation in independent knockout clones as only one validated clone was analyzed.

DNA methylation plays a central role in shaping the transcriptional landscape of gliomas, influencing both tumor classification [35] and the functional heterogeneity that defines GBM [36,37]. We found that *CKAP2L* expression may be regulated through epigenetic mechanisms in glioma. The CpG site cg00908571 within *CKAP2L* promoter region showed progressive hypomethylation across glioma grades which strongly correlated with increased *CKAP2L* expression. This observation suggests that cg00908571 may represent a potential regulatory site.

Collectively, these findings indicate that *CKAP2L* plays an important role in supporting glioma cell proliferation, clonogenicity and migration by regulating mitotic progression and cell motility rather than cell survival. The functional consequences observed upon CKAP2L deletion complement the transcriptomic analyses linking *CKAP2L* expression to cell-cycle and oncogenic programs, important for maintaining proliferative and invasive tumor cell states in glioblastoma.

## Data Availability

The raw and processed RNA-seq data generated during this study have been deposited in the Gene Expression Omnibus (GEO) under accession number GSE350713. All other relevant data are included within the article or its Supplementary Information Files.

## Supplementary Materials

The following supporting information can be downloaded at: https://www.mdpi.com/article/10.3390/cells15211996/s1, Figure S1: Gene Ontology enrichment analysis of differen-tially expressed genes in early-stage tumors; Figure S2: Gene Ontology enrichment analysis of differentially expressed genes in advanced-stage tumors; Figure S3: siRNA screening identifies candidate regulators of glioblastoma cell proliferation and migration; Figure S4: IPA network and gene ontology enrichment analyses of CKAP2L associated genes; Figure S5: CKAP2L expression in TCGA gliomas; Figure S6: CKAP2L expression and promoter DNA methylation in TCGA gliomas; Table S1: Target sequences of the siRNA screening; Table S2: Differentially expressed genes in tumor versus non-tumor olfactory bulbs; Table S3: Differen-tially expressed genes in advanced versus early tumors; Table S4: Pathway enrichment from IPA for activated and inhibited pathways at early tumor versus non tumor control; Table S5: Pathway enrichment from IPA for activated and inhibited pathways at advanced tumor versus early tumor; Table S6: Functional annotation of gene clusters of RNA-seq data; Table S7: Normalized cell count for the high-content siRNA screening; Table S8: Upstream regulators from IPA for activated and inhibited pathways at early tumor versus non tumor control and advanced vs early tumor RNAseq Data.
