# Supplementary material for: CKAP2L Promotes Proliferation and Migration of Glioma Cells in a DIAPH3-Deficient Model
Source: Cells. 2026 Oct 31;15(21):1996. doi: 10.3390/cells15211996 (PMC13761541; doi:10.3390/cells15211996)
Supplement: Supplementary file 1 [file cells-15-01996-s001.zip › Cells-4593487-Supplementary Information.pdf]

CKAP2L Promotes Proliferation and Migration of Glioma Cells in a DIAPH3-Deficient Model

Asma Mahdi [1], Arwa Aldaalis [1], Georges Chehade [2], Eva On-Chai Lau [2], Hend Salah Fayed [1], Ayman Al Haj Zen [1], Mohamed Aittaleb [1], Maisa Ismael Alkailani [1] and Fadel Tissir [1,*]

[1] College of Health and Life Sciences, Hamad Bin Khalifa University, Penrose House Building, Education City, Doha P.O. Box 34110, Qatar; asmamahdi.contact@gmail.com (A.M.); aaldaalis@hbku.edu.qa (A.A.); hend.fayed.91@gmail.com (H.S.F.); aalhajzen@hbku.edu.qa (A.A.H.Z.); maittaleb@hbku.edu.qa (M.A.); malkailani@hbku.edu.qa (M.I.A.)

[2] Developmental Neurobiology Laboratory, Institute of Neuroscience, Université Catholique de Louvain,
1200 Brussels, Belgium; georges.chehade@uclouvain.be (G.C.); onchaii@hotmail.com (E.O.-C.L.)

* Correspondence: ftissir@hbku.edu.qa; Tel.: +974-508-123-19

# This file contains supplementary materials and methods, and six supplementary figures and their legends.

Supplementary Materials and Methods

RNA sequencing

For RNA sequencing, tumor samples were obtained from a genetically engineered mouse model generated by conditional deletion of Diaph3 and Trp53 in dorsal telencephalic neural progenitors using the Emx1-Cre driver [1]. Mice were monitored using an ultra-high-resolution (7-Tesla) MRI scanner. Early-stage tumors ($\leq$10 mm^3) located in the olfactory bulb, and advanced tumors ($\geq$50 mm^3) extending from the olfactory bulb to the frontal cortex were dissected for RNA-seq. For early-stage tumors, matched non-tumor tissue was collected from the contralateral olfactory bulb. Three biological replicates were analyzed per group. No samples were excluded from the analysis.

Total RNA was extracted from dissected brain tissues using the ReliaPrepTM RNA Miniprep System (Promega). Ribodepletion was carried out to remove ribosomal RNA and enrich in messenger RNA prior to library preparation. RNA quantity and integrity were assessed by fluorometric measurement and electrophoretic profiling. Strand-specific cDNA libraries were generated using the Illumina TruSeq Stranded RNA workflow on an automated system. After adapter ligation and PCR amplification, the resulting libraries were subjected to standard quality control procedures, pooled, and sequenced on the Illumina HiSeq 2500 platform using paired-end chemistry.

Raw sequencing files were examined using FastQC to assess base quality scores, GC composition, and potential sequencing artifacts. Adapter sequences and low-quality bases were removed using Trimmomatic, leaving only high-quality reads for further analysis.

Filtered reads were aligned to the mouse reference genome (GRCm38/mm10) using the splice-aware aligner HISAT2. Gene-level quantification was performed using featureCounts to generate a count matrix for all samples. Differential expression analysis was conducted in DESeq2, including size-factor normalization and dispersion estimation. Two comparisons were conducted: early tumors ($<10\ mm^3$) versus matched non-tumor controls, and advanced tumors ($\geq 50\ mm^3$) versus early tumors. Genes with $|\log_2$ fold-change$| \geq 1$ and adjusted p-value ≤ 0.05 were considered significantly differentially expressed. Pseudogenes and long non-coding RNAs were excluded. Principal component analysis (PCA) was performed to visualize sample clustering across tumor stages.

Functional enrichment and pathway analysis

To functionally annotate the transcriptional changes observed across tumor stages, pathway-level interpretation was performed using the Ingenuity Pathway Analysis (IPA) software (QIAGEN Inc., https://digitalinsights.qiagen.com) to identify significantly enriched canonical pathways and to predict upstream regulators based on submitted fold-change and *p*-value data. Pathway significance was evaluated using activation Z-scores and *p*-values of overlap. Gene Ontology (GO) enrichment analysis was performed on the sets of significantly upregulated and downregulated genes using SRPlot (https://www.bioinformatics.com.cn/en) focusing on the GO Biological Process (BP), Molecular Function (MF), and Cellular Component (CC) categories. Cluster-based analyses, including k-means clustering performed with iDEP (iDEP.96) (https://bioinformatics.sdstate.edu/idep96/) were used to group genes with similar expression trajectories across no-tumor samples, early-stage tumors, and advanced-stage

tumors.

TCGA data acquisition and analysis

To assess the relevance of selected genes in human glioma, the Cancer Genome Atlas (TCGA) [2] data were accessed through different online platforms, each used for a specific analytical purpose. Correlation analyses were performed using GEPIA (Gene Expression Profiling Interactive Analysis; http://gepia.cancer-pku.cn). GEPIA provides normalized RNA-seq expression values derived from TCGA and normal brain tissues derived from the Genotype-Tissue Expression (GTEx) project [3]. Correlation coefficients were generated using the built-in Pearson correlation module; scatter plots were obtained directly from the platform. Associations between gene expression and overall survival in patients with glioblastoma were evaluated using OncoLnc (www.oncolnc.org) and Gliovis (https://gliovis.bioinfo.cnio.es). Patients were stratified into high- and low-expression groups using the platform's median-based cutoff, and Kaplan–Meier survival curves with log-rank *p*-values were generated. No additional statistical adjustments were applied beyond the tool's default settings. Expression variation and methylation levels across glioma grades (WHO grades II–IV) were explored using the UCSC Xena Browser (https://xenabrowser.net). The "TCGA LGG/GBM" cohort of 1153 patients was used to extract normalized expression values and beta values for comparisons across tumor grades. No additional normalization was applied beyond the standardized TCGA preprocessing provided by the platform. Gene set enrichment analysis (GSEA) was performed using the GSEA software developed by the Broad Institute [14]. Patients were stratified into CKAP2L-high and CKAP2L-low groups using the median CKAP2L expression level as the cutoff. The analysis was conducted on 702 samples, comprising 337 samples in the

CKAP2L-high group and 365 in the CKAP2L-low group. Gene expression profiles were ranked according to their association with the two phenotypes, and enrichment was evaluated using gene sets from the Molecular Signatures Database (MSigDB), including the Hallmark and oncogenic signature collections. Gene sets containing 15–500 genes were included. Enrichment was assessed using the normalized enrichment score (NES), nominal *p*-value, and false discovery rate (FDR) q-value. An FDR q-value below 0.25 was used as the significance threshold for GSEA, consistent with the conventional threshold for exploratory gene set enrichment analysis.

To investigate the association between TP53 mutation status and CKAP2L expression, RNA-sequencing, mutation and clinical data were obtained from the TCGA PanCancer Atlas GBM and LGG studies through cBioPortal (gbm_tcga_pan_can_atlas_2018 and lgg_tcga_pan_can_atlas_2018). CKAP2L and MKI67 expression values were obtained as batch-normalized RSEM values and transformed using $\log_2$(RSEM + 1). TP53, IDH1, and IDH2 mutation data were used to classify tumors by mutation status. Following the relevant exclusions, 668 patients were included in the expression analysis (154 GBM and 514 LGG). Two-group comparisons were performed using unpaired two-tailed Student's t-tests and comparisons among multiple groups using the Kruskal–Wallis test as appropriate.

Cox proportional hazards regression was performed in R using the survival package to assess the association between CKAP2L expression and OS. The final survival cohort comprised 564 patients (373 IDH-mutant and 191 IDH-wildtype). Univariate and multivariable models were fitted adjusting for the clinical and molecular covariates included in each model with additional analyses stratified by IDH status. Results were

reported as hazard ratios (HRs) with 95% confidence intervals (CIs) and p-values. Statistical significance was set at $p < 0.05$.

Cell culture

U-251 MG human glioblastoma cells (U251) were used throughout this study. Cells were maintained in Dulbecco's Modified Eagle Medium (DMEM; Gibco, DMEM (1X) + GlutaMAX™-I) supplemented with 10% heat-inactivated fetal bovine serum (FBS; Gibco) and 1% Penicillin-Streptomycin (Gibco, 15140-122). Cultures were incubated at 37°C in a humidified atmosphere containing 5% CO_2. Three cell populations were used: U251 control (CTL), U251 DIAPH3 knockout (DIAPH3 KO), and U251 CKAP2L knockout (CKAP2L KO).

Generation of DIAPH3 and CKAP2L Knockout Cell Lines

Stable DIAPH3 and CKAP2L knockout U251 cell lines were generated using the Edit-R All-in-One Lentiviral sgRNA System (Horizon Discovery) according to the manufacturer's instructions. U251 cells were seeded in 6-well plates at 1.2 × 10^5 cells/well in 2 mL of growth medium and incubated overnight at 37 °C with 5% CO_2. Cells were subsequently transduced with Edit-R lentiviral sgRNA particles targeting DIAPH3 (VSGH11936; 10^7 TU/mL) or CKAP2L (VSGH12606; 10^7 TU/mL), carrying Cas9, the gene-specific sgRNA and a puromycin-resistance cassette under the mCMV promoter. A non-targeting lentiviral control (VSGC11954; 10^7 TU/mL) was used to generate the control population. Following transduction at an MOI of 0.3, cells were allowed to recover and subsequently subjected to puromycin selection to enrich for successfully transduced cells. Selection medium was replaced every 2–3 days, and cells

were monitored until non-transduced cells were eliminated and resistant cells resumed normal growth. Puromycin-resistant cells were then expanded and individual clones were isolated. Candidate clones were validated by Sanger sequencing at the HBKU Core Labs to confirm CRISPR/Cas9-mediated editing at the target locus, followed by Western blotting to assess the corresponding protein expression. Clones showing confirmed gene editing with reduced or absent DIAPH3 or CKAP2L protein expression were selected for subsequent experiments.

Cell-cycle analysis by flow cytometry

U251 CTL and CKAP2L KO cells were collected, washed with phosphate-buffered saline (PBS) and fixed by slowly resuspending the pellets in ice-cold 70% ethanol while vortexing. Fixed cells were washed with 0.5% BSA (Thermo Fisher Scientific, Albumin Standard, 23209) in PBS to remove residual ethanol, and pellets were resuspended in a cold staining solution containing 50 µg/mL Propidium Iodide (PI) and 25 µg/mL RNase A. Samples were incubated protected from light for 1 hour at room temperature to allow complete RNA digestion and PI incorporation. Samples were acquired on the BD Accuri C6 Plus Flow Cytometer (BD Biosciences).

Apoptosis analysis

Apoptosis was assessed using the CellEvent™ Caspase-3/7 Green Detection Reagent (Invitrogen, C10427) according to the manufacturer's instructions with minor modifications. U251 CTL and CKAP2L KO cells were collected by trypsinization and centrifugation and washed in 0.2% BSA in PBS and stained with the CellEvent™ Caspase-3/7 Green Detection Reagent at a dilution of 1:1000. Samples were incubated for 1h at room temperature in the dark. During the last 5 minutes of incubation, a dead-

cell stain (SYTOX™ AADvanced Dead Cell Stain) was added to exclude non-viable cells. After staining, samples were centrifuged and resuspended in wash buffer and analyzed on the Beckman Coulter CytoFLEX (3 Laser system) flow cytometer. Data were analyzed using FlowJo software.

Wound-healing assay

Wound-healing assays were performed using the Culture-Insert 2 Well in μ-Dish 35 mm (ibidi). U251 CTL and CKAP2L KO cells were seeded into the insert chambers and allowed to form a confluent monolayer. After insert removal, cells were maintained in a low-serum medium (0.5% FBS) to minimize the contribution of cell proliferation to wound closure. Images of the wound area were acquired immediately following insert removal (0 h) and every 24 h. Wound closure was quantified by measuring the remaining wound area using ImageJ analysis software.

Western blotting

Cells were lysed on ice in RIPA buffer supplemented with 1× protease and phosphatase inhibitor cocktails. Protein concentration was determined using the Pierce BCA Protein Assay Kit (Thermo Fisher Scientific) according to the manufacturer's instructions, with absorbance measured using the NanoDrop spectrophotometer. Samples were adjusted to 10 μg total protein per lane, mixed with 4× loading buffer and denatured at 95 °C before separation by 4–12% precast SDS–PAGE gels (Invitrogen), using NuPAGE™ MES SDS Running Buffer (20×; NP0002). Proteins were transferred onto PVDF membranes using Tris-glycine transfer buffer prepared from 10× Tris-Glycine SDS Buffer (28362), methanol, and ultrapure water. Membranes were blocked with 5% (w/v) BSA in PBST for 1 h at room temperature and incubated overnight at 4 °C with primary antibodies

diluted in 5% BSA/PBST. The primary antibodies used were anti-CKAP2L (Proteintech, 17143-1-AP; 1:1000) and anti-GAPDH (abcam, ab9485, 1:5000). After washing with PBST, membranes were incubated with the appropriate HRP-conjugated secondary antibodies for 1 h at room temperature. Chemiluminescent signals were developed using Clarity™ Western ECL Substrate (Bio-Rad, 1705060) and captured using the iBright™ CL1500 Imaging System. Band intensities were quantified using ImageJ software and normalized to the corresponding GAPDH signal.

Statistical analysis

For the transcriptomic data, differential gene expression significance was determined using p adjusted (Padj) values derived from the internal statistical frameworks of the bioinformatic tools employed (DESeq2-based workflows). Additional pathways and enrichments analyses were performed using software packages that include their own embedded statistical algorithms. For in vitro experiments, statistical analyses were performed using the GraphPad Prism 8. Depending on the experimental design, one-way ANOVA, two-way ANOVA, or unpaired two-tailed Student's t-tests were applied as specified in the figure legends. Data are presented as mean ± standard error of the mean (SEM), and statistical significance was defined as $p < 0.05$. The levels of significance were obtained as follows: * $p < 0.05$, ** $p < 0.01$, *** $p < 0.001$.

Supplementary discussion

Supplementary Figures and Legends

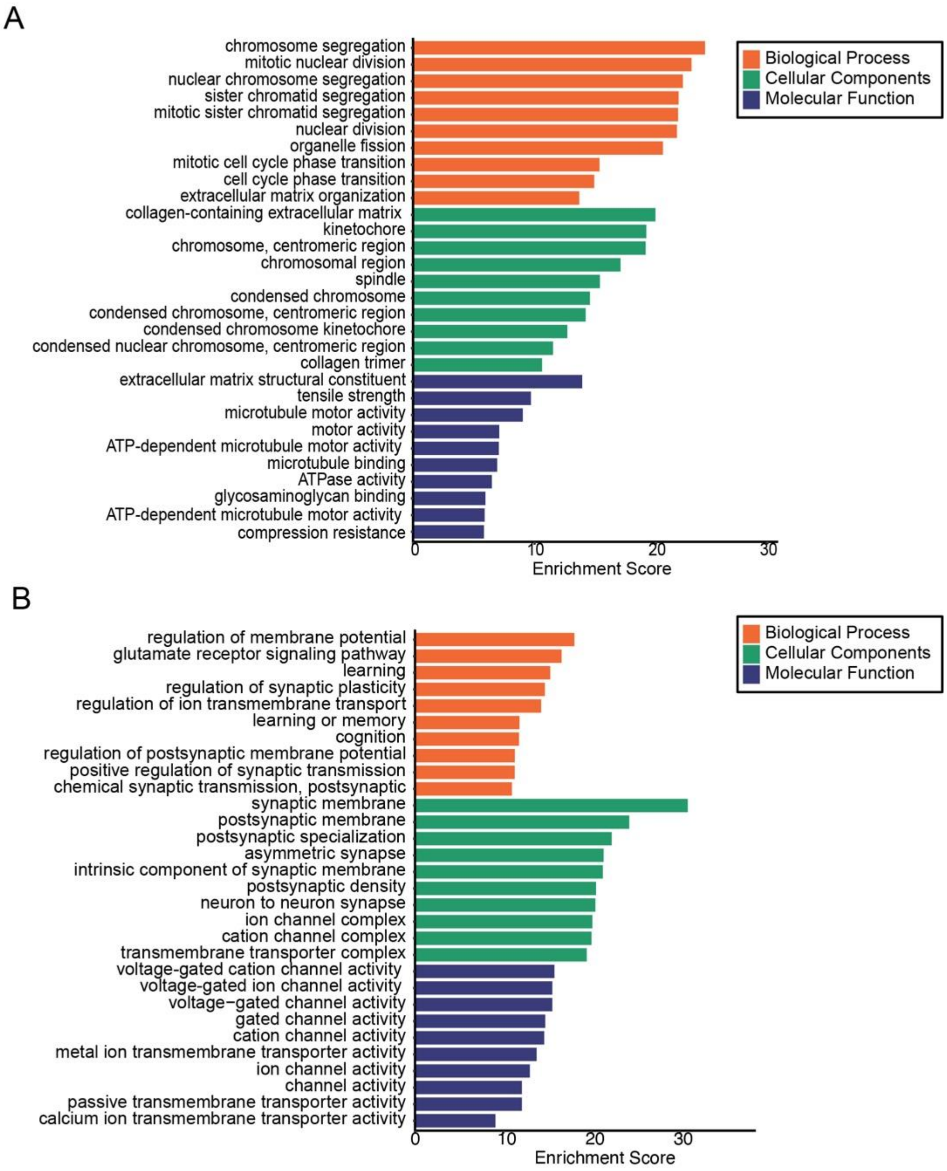

Supplementary Figure S1: Gene Ontology enrichment analysis of differentially expressed genes in early-stage tumors.

(A) Top 10 enriched Biological Process, Molecular Function, and Cellular Component GO terms among upregulated genes (UR) in early-stage tumors versus controls. (B) Top 10 enriched Biological Process, Molecular Function, and Cellular Component GO terms among downregulated genes (DR) in early-stage tumors versus controls.

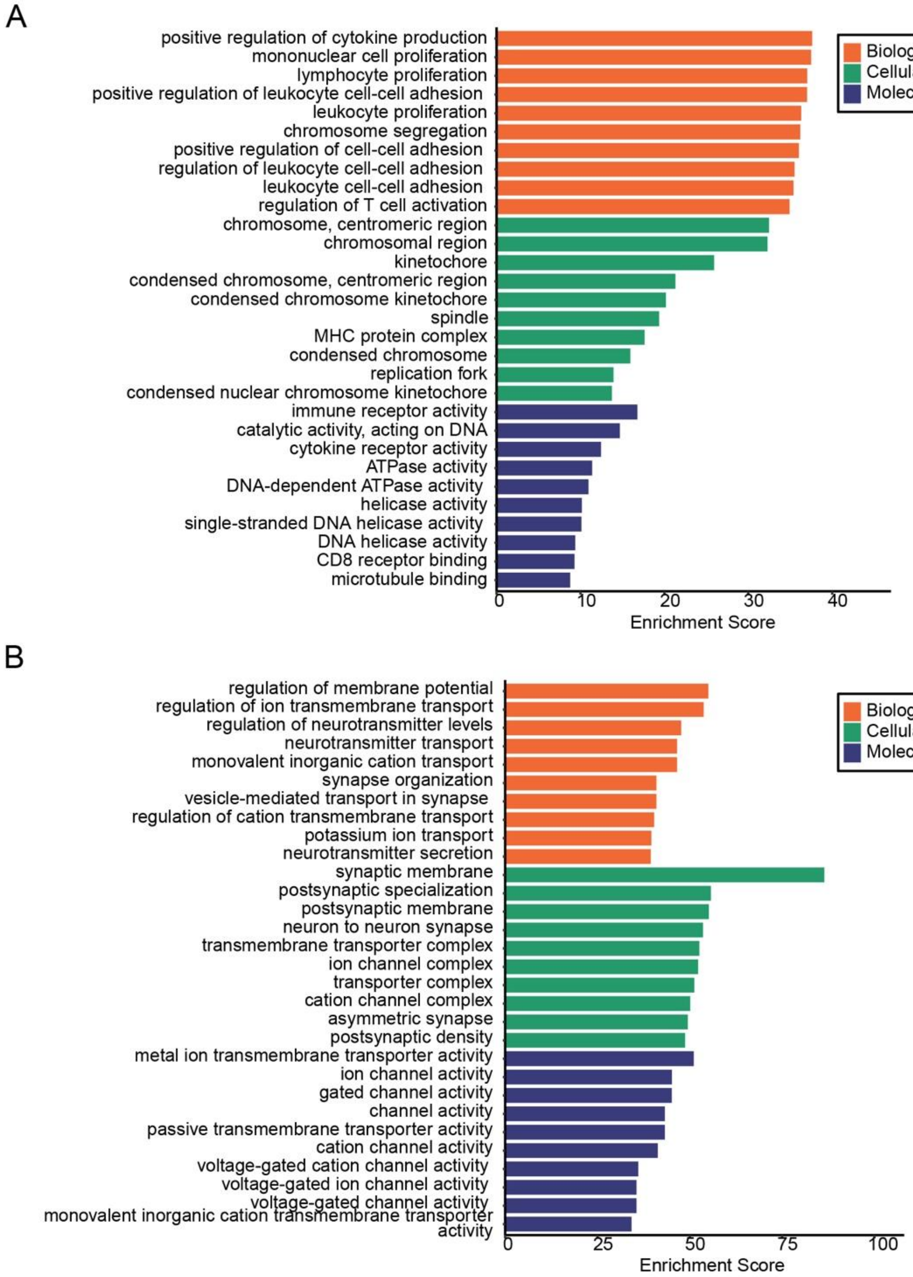

Supplementary Figure S2. Gene Ontology enrichment analysis of differentially expressed genes in advanced-stage tumors.

(A) Top 10 enriched Biological Process, Molecular Function, and Cellular Component GO terms among upregulated genes in advanced-stage vs early-stage tumors. (B) Top 10 enriched Biological Process, Molecular Function, and Cellular Component GO terms among downregulated genes in advanced-stage vs early-stage tumors.

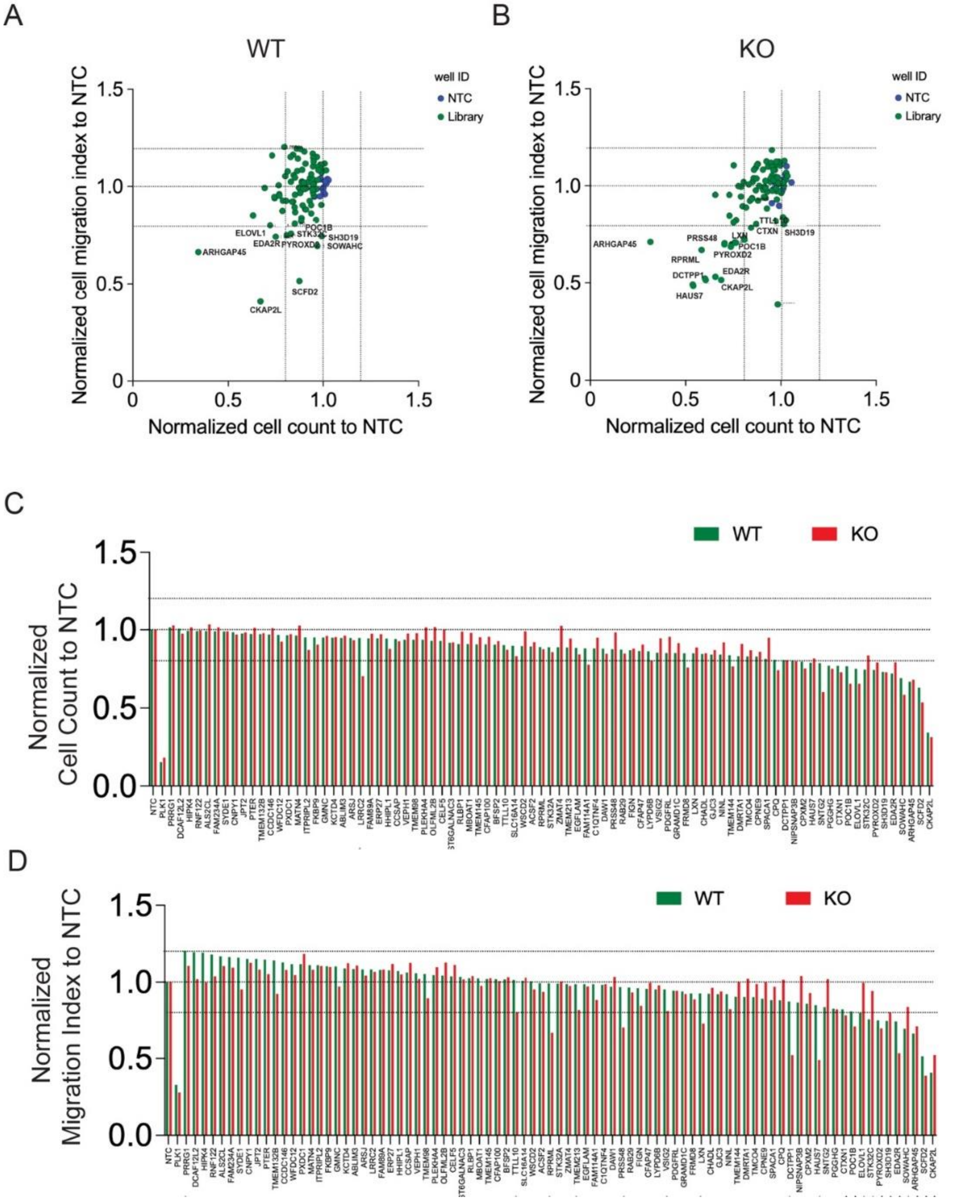

Supplementary Figure S3: siRNA screening identifies candidate regulators of glioblastoma cell proliferation and migration.

(A-B) Integrated analysis of migration in WT (A) and KO (B) cells. Genes located in the lower-left quadrant exhibited concurrent reductions in cell count and migration, highlighting CKAP2L as a prominent regulator of glioblastoma cell growth and motility. (C) Ranked distribution of normalized cell counts for all screened siRNAs in CTL (green) and DIAPH3 KO (red) cells. (D) Ranked distribution of normalized migration index for all screened siRNAs in CTL (green) and KO (red) cells. Across panels, each bar represents one siRNA condition, and all measurements are normalized relative to the NTC within each genotype.

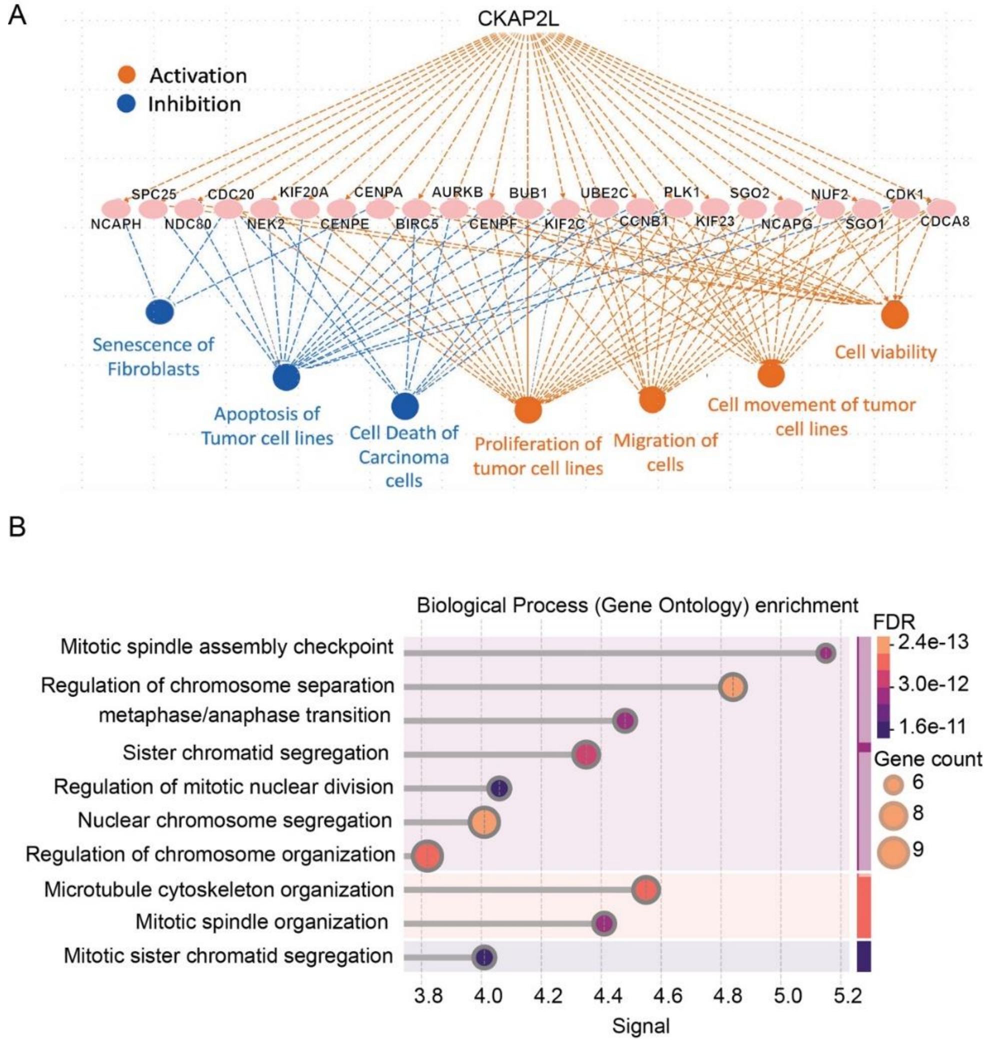

Supplementary Figure S4: IPA network and Gene Ontology enrichment analyses of CKAP2L-associated genes.

(A) IPA-based functional interaction network illustrating downstream biological functions associated with CKAP2L, based on input gene expression data and curated knowledge from the Ingenuity Pathway Analysis database. (B) GO enrichment analysis of the associated genes showing the top 10 enriched biological processes.

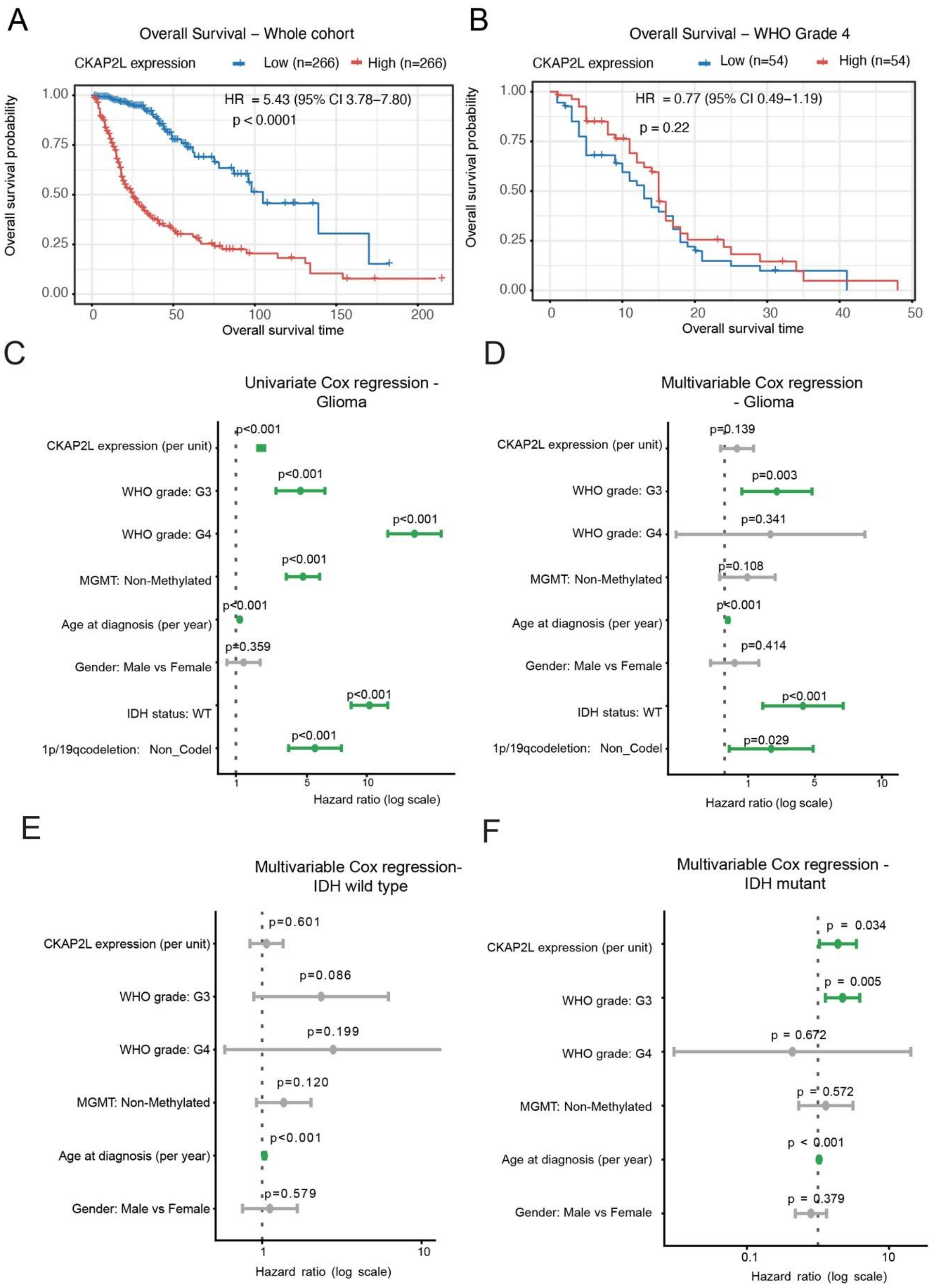
A
Overall Survival – Whole cohort
CKAP2L expression
Low (n=266)
High (n=266)
HR = 5.43 (95% CI 3.78–7.80)
p < 0.0001
Overall survival probability
Overall survival time
B
Overall Survival – WHO Grade 4
CKAP2L expression
Low (n=54)
High (n=54)
HR = 0.77 (95% CI 0.49–1.19)
p = 0.22
Overall survival probability
Overall survival time
C
Univariate Cox regression - Glioma
D
Multivariable Cox regression - Glioma
CKAP2L expression (per unit)
WHO grade: G3
WHO grade: G4
MGMT: Non-Methylated
Age at diagnosis (per year)
Gender: Male vs Female
IDH status: WT
1p/19qcodeletion: Non_Codel
Hazard ratio (log scale)
E
Multivariable Cox regression- IDH wild type
F
Multivariable Cox regression - IDH mutant
Hazard ratio (log scale)

Supplementary Figure S5: CKAP2L expression in TCGA gliomas.

(A) Kaplan–Meier survival analysis of all glioma patients from the TCGA cohort (LGG + GBM). (B) Kaplan–Meier survival analysis of GBM from the TCGA cohort. (C-F) Cox regression analysis in TCGA glioma cohort (LGG + GBM). (C) Univariate Cox regression analysis; CKAP2L: HR = 1.56 (95% CI: 1.47–1.66), $p < 0.001$; WHO grade G3: HR = 3.12 (2.03–4.79), $p < 0.001$; WHO grade G4: HR = 23.14 (14.49–36.97), $p < 0.001$; MGMT non-methylated: HR = 3.26 (2.43–4.37), $p < 0.001$; Age: HR = 1.07 per year (1.06–1.08), $p < 0.001$; Male vs female: HR = 1.15 (0.86–1.54), $p = 0.359$; IDH-wild type: HR = 10.47 (7.59–14.44), $p < 0.001$; 1p/19q non-codeleted: HR = 4.02 (2.53–6.40), $p < 0.001$. (D) Multivariate Cox regression analysis; CKAP2L: HR = 1.17 (95% CI: 0.95–1.45), $p = 0.139$; WHO grade G3: HR = 1.96 (1.25–3.09), $p = 0.003$; WHO grade G4: HR = 1.81 (0.54–6.09), $p = 0.341$; MGMT non-methylated: HR = 1.34 (0.94–1.92), $p = 0.108$; age: HR = 1.04 per year (1.03–1.06), $p < 0.001$; Male vs female: HR = 1.14 (0.83–1.55), $p = 0.414$; IDH-wild type: HR = 2.75 (1.64–4.61), $p < 0.001$; 1p/19q non-codeleted: HR = 1.82 (1.06–3.13), $p = 0.029$. (E) Multivariable Cox regression in IDH-wild-type gliomas (n = 190; 122 events); CKAP2L: HR = 1.07 (95% CI: 0.84–1.35), $p = 0.601$; WHO grade G3: HR = 2.34 (0.89–6.20), $p = 0.086$; WHO grade G4: HR = 2.79 (0.58–13.32), $p = 0.199$; MGMT non-methylated: HR = 1.37 (0.92–2.02), $p = 0.120$; Age: HR = 1.03 per year (1.02–1.05), $p < 0.001$; Male vs female: HR = 1.12 (0.75–1.66), $p = 0.579$. (F) Multivariable Cox regression in IDH-mutant gliomas (n = 373; 65 events); CKAP2L: HR = 1.91 (95% CI: 1.05–3.48), $p = 0.034$; WHO grade G3: HR = 2.22 (1.27–3.88), $p = 0.005$; WHO grade G4: HR = 0.44 (0.009–20.27), $p = 0.672$; MGMT non-methylated: HR = 1.29 (0.54–3.10), $p = 0.572$; Age: HR = 1.04 per year (1.02–1.06), $p < 0.001$; Male vs female: HR = 0.80 (0.48–1.32), $p = 0.379$. In panels C-F, points represent HRs, horizontal bars indicate 95% confidence intervals (CIs), and the vertical dotted line indicates HR = 1. The x-axis is logarithmic. Green indicates $p < 0.05$; gray indicates $p \geq 0.05$. All p-values are two-sided.

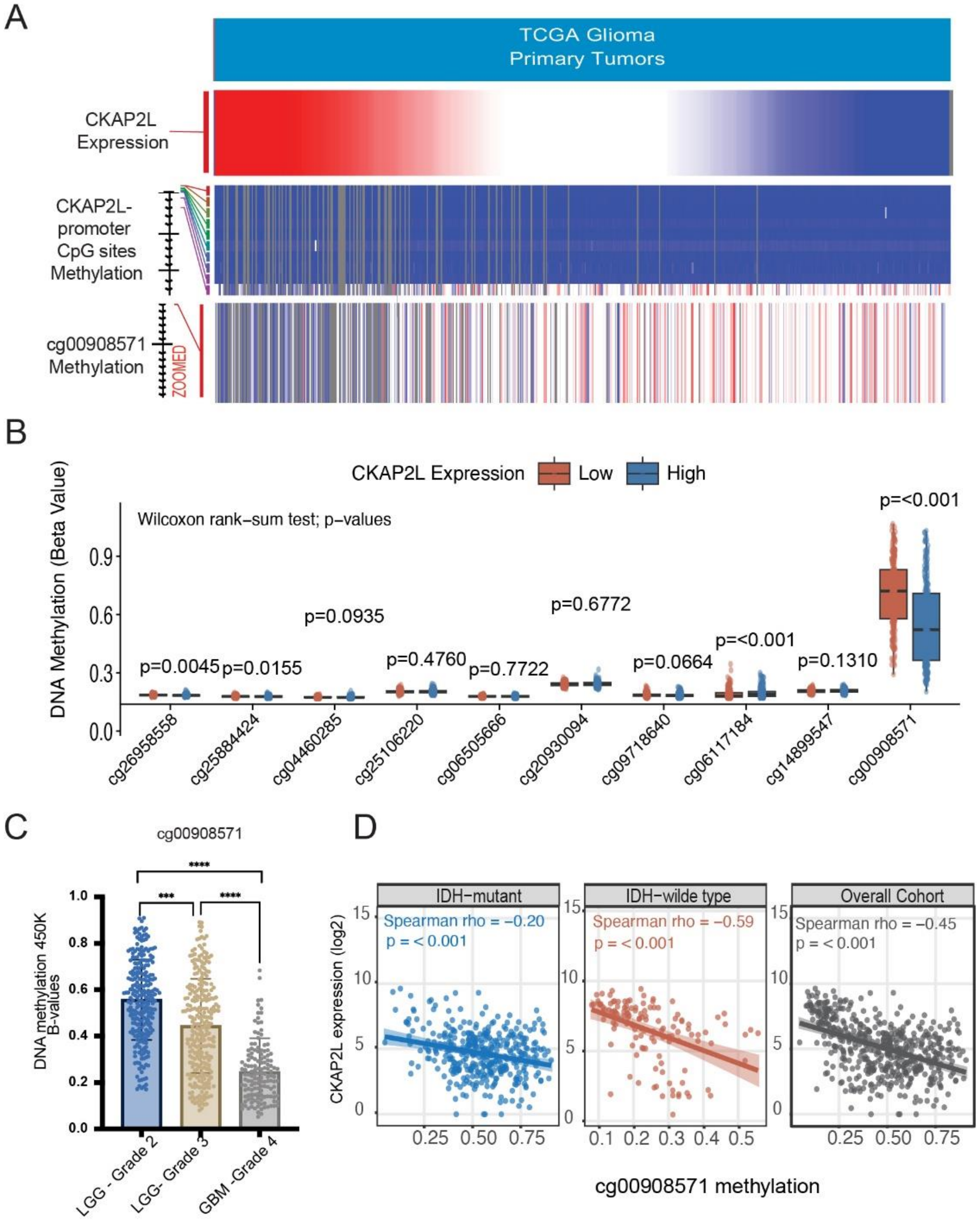

Supplementary Figure S6: CKAP2L expression and promoter DNA methylation in TCGA gliomas.

(A) Samples from TCGA glioma primary tumors are ranked according to CKAP2L expression (left). The middle panel shows methylation levels across CpG sites within the CKAP2L promoter region, while the right panel presents a zoomed view of CpG probe cg00908571. (B) Promoter CpG sites methylation β-values between low- and high-CKAP2L expression groups. (C) β-value distributions of cg00908571 across LGG and GBM samples from TCGA. (D) Spearman correlation between cg00908571 methylation and CKAP2L expression in IDH-mutant gliomas (n = 377), IDH-wild-type gliomas (n = 130), and the overall cohort (n = 507).

Supplementary References

1. Chehade, G.; Durá, I.; Ruiz-Reig, N.; Damiani, D.; Lau, E.O.-C.; Lelotte, J.; Joudiou, N.; Aittaleb, M.; Tissir, F. Loss of DIAPH3 accelerates glioma genesis in mice. *Cell Death & Disease* **2026**.
2. Weinstein, J.N.; Collisson, E.A.; Mills, G.B.; Shaw, K.R.; Ozenberger, B.A.; Ellrott, K.; Shmulevich, I.; Sander, C.; Stuart, J.M. The cancer genome atlas pan-cancer analysis project. *Nature genetics* **2013**, *45*, 1113-1120.
3. Lonsdale, J.; Thomas, J.; Salvatore, M.; Phillips, R.; Lo, E.; Shad, S.; Hasz, R.; Walters, G.; Garcia, F.; Young, N. The genotype-tissue expression (GTEx) project. *Nature genetics* **2013**, *45*, 580-585.
